# Effect of road transport for up to 24 hours followed by twenty-four hour recovery on live weight and physiological responses of bulls

**DOI:** 10.1186/1746-6148-6-38

**Published:** 2010-07-20

**Authors:** Bernadette Earley, Margaret Murray, Daniel J Prendiville

**Affiliations:** 1 Animal and Bioscience Research Department, Animal & Grassland Research and Innovation Centre, Teagasc, Grange, Dunsany, Co. Meath, Ireland

## Abstract

**Background:**

The transport of livestock can have major implications for their welfare, and there is strong public interest and scientific endeavour aimed at ensuring that the welfare of transported animals is optimal. The objective of the study was to investigate the effect of transport on live weight, physiological and haematological responses of bulls after road transport of 0, 6, 9, 12, 18 and 24 hours (h). Seventy-two Charolais bulls (mean weight (s.d.) 367 (35) kg), naïve to transport, were randomly assigned to one of six journey (J) times of 0 h, 6 h, 9 h, 12 h, 18 h and 24 h transport (n = 12 animals/treatment) at a stocking density of 1.02 m^2^/bull. Blood samples were collected by jugular venipuncture before transport (-0.25 h), immediately after (0 h) and at 1 h, 2 h, 4 h, 6 h, 8 h, 12 h and 24 h relative to time 0 h. The bulls were weighed before transport (- 24 h and - 0.25 h), immediately after (0 h), and at 4 h, 12 h and 24 h relative to time 0 h. Control animals were blood sampled before assignment (-0.25 h) to novel pens, after (24 h), and at 1 h, 2 h, 4 h, 6 h, 8 h, 12 h and 24 h relative to the 24 h sampling time point.

**Results:**

Bulls travelling for 6 h (280 km), 9 h (435 km), 12 h (582 km), 18 h (902 km) and 24 h (1192 km) lost 4.7, 4.5, 5.7 (P < 0.05), 6.6 (P < 0.05) and 7.5 (P < 0.05) percentage (%) live weight compared with baseline. Live weight re-gained to pre-transport levels during the 24 h recovery period. Lymphocyte percentages were lower (P < 0.05) and neutrophil percentages were greater (P < 0.05) in all animals. Blood protein, glucose and NEFA concentrations and creatine kinase activity were greater (P < 0.05) in the bulls following transport and returned to baseline within 24 h.

**Conclusions:**

Under the conditions of the present study, transport of bulls on journeys by road, ranging from 6 h (280 km) to 24 h (1192 km) duration, affected live weight, haematological and physiological measurements of metabolism and inflammation. Our findings showed that live weight and some physiological and haematological responses of bulls returned to pre-transport levels within 24 h with animals having had access to feed and water.

## Background

Transportation of livestock involves several potential stressors that result in increased cortisol [1-7], mobilisation of energy and protein metabolism [7], and a challenged immune system [8-12] resulting in increased disease susceptibility [13]. Studies have been carried out to determine the optimum stocking density, the maximum duration of transportation, the timing of rest stops and which components of the transport process are the most stressful to cattle [3,4,14-17]. Physical factors such as noise or vibrations; psychological/emotional factors, such as unfamiliar environment or social regrouping; and climatic factors, such as temperature and humidity, are also involved in the transport process. Steers transported (12-18 mo old) by road for 5, 10 and 15 h lost 4.6, 6.5 and 7.0% of their live weight; and recovery to pre-transport live weight took 5 days [18,19]. There are limited scientific data on the physiological and haematological recovery of animals after long durations of transport and in particular the physiological recovery of animals during the 24 h period post-transport.

The objective of the study was to evaluate the effect of transport on live weight, physiological and haematological responses of bulls after road transport of 0, 6, 9, 12, 18 and 24 h and on their physiological recovery during the 24 h period post-transport.

## Results

### Environment

The mean temperature of the shed environment where the animals were housed was 15.6°C (minimum 6.0°C and maximum 19.3°C). The environmental data recorded during each transport journey are reported in Table 1. The levels of CO_2 _tended to increase with the longer journeys and values were numerically greater during the 24 h journey compared with the other journeys. The levels of H_2_S and NH_3 _remained low throughout the series of transport journeys. Vapour density and ambient temperature during the 9 h transport journey (J 9) was numerically greater compared with the other journey durations.

**Table 1:** Environmental conditions recorded during housing and during transport.

Transport journeys	Ambient Temperature (°C)	Environmental conditions recorded during housing of control (J 0) and during transport of J 6, J 9, J 12, J18 and J 24 treatments						
		
		**CO**_**2**_ (ppm)	Relative humidity (%)	Temperature (°C)	Wind speed (m/s)	Vapour density (g/m^3^)	H_2_S (ppm)	**NH**_**3**_ (ppm)
J 0	12.0 (1.7-20.2)	471.3 (417.0-655.0)	69.8 (40.9-88.6)	15.6 (6.0-19.0)	0.4 (0.0-0.10)	4.3 (2.8-8.6)	0.00 (0.00-0.05)	0.0 (0.0-0.0)
J 6	8.4 (6.1-10.3)	453.8 (356.0-897.0)	89.5 (72.5-99.9)	7.6 (4.7-10.8)	0.8 (0.1-2.5)	5.9 (4.3-10.8)	0.01 (0.00-0.10)	1.3 (0.0-5.7)
J 9	15.3 (11.3-17.7)	531.6 (388.0-922.0)	93.8 (81.1-99.9)	14.4 (10.4-17.7)	0.6 (0.0-2.3)	13.4 (10.4-15.9)	0.00 (0.00-0.00)	0.4 (0.0-2.9)
J 12	7.8 (0.7-14.2)	668.8 (447.0-1903.0)	74.7 (61.5-89.2)	8.6 (3.5-12.6)	0.7 (0.0-3.0)	4.3 (1.2-8.7)	0.01 (0.00-0.20)	0.9 (0.0-7.7)
J 18	7.1 (3.5-9.9)	545.5 (399.0-858.0)	78.1 (65.0-91.0)	8.3 (4.7-10.5)	0.8 (0.0-3.3)	4.6 (0.5-8.5)	0.04 (0.00-0.20)	0.0 (0.0-0.0)
J 24	4.9 (-3.1-18.1)	770.9 (401.0-2373.0)	86.2 (64.3-99.9)	6.4 (-0.1-16.0)	0.6 (0.0-3.1)	4.2 (-1.1-12.2)	0.00 (0.00-0.00)	0.1 (0.0-2.9)

The values are expressed as mean with minimum and maximum values.

Control = J 0 not transported (control); J 6, J 9, J 12, J 18 and J 24 = animals transported for journey durations of 6, 9, 12, 18 and 24 hours (h) by road at a stocking density of 1.02 m^2 ^per animal.

### Water intake

Table 2 shows the consumption of water during and after transport. Most of the transported treatments consumed water during the transport journeys except for the J 12 treatment which did not consume water during transport. Post-transport all treatment groups had numerically greater water intake volumes in the 1 - 4 h period post-transport.

**Table 2:** Water consumption (litres) per animal (n = 12 animals per treatment) during and after different transport journey (J) durations.

J duration (hour (h))	Water intake (litres) during transport	Distance travelled by road (km)	Post-transport (water intake (litres))		
			
			0 - 4 h	5 - 12 h	13 - 24 h
0 h	4.1	0	20.2^a^	4.5^b^	0.0^b^
6 h	5.0	280	8.0^a^	3.5^b^	0.5^b^
9 h	5.0	435	11.5^a^	1.5^b^	6.0^b^
12 h	0.0	582	8.5^a^	6.5^ab^	4.0^b^
18 h	1.8	902	5.5^a^	7.0^ab^	3.0^b^
24 h	3.9	1192	9.0^a^	7.0^ab^	1.0^c^

Control = J 0 not transported (control); J 6, J 9, J 12, J 18 and J 24 = animals transported for journey durations of 6, 9, 12, 18 and 24 hours by road at a stocking density of 1.02 m^2 ^per animal.

^a,b, ^within row means not having a common superscript differ significantly (P < 0.05).

### Live weight

Significant (P < 0.05) live weight loss was observed for the J 18 and J 24 treatments and only at 0 h post-transport (Table 3). Bulls travelling for 6, 9, 12, 18 and 24 h lost 4.6, 4.5, 5.7, 6.3 and 7.5% live weight (P < 0.05) compared with baseline while control animals lost 5.4%. During the 24 h recovery period live weight was regained to pre-transport levels in all treatments at 4 h (Table 3).

**Table 3:** Mean live weight (kg) ± s.d. in control and transported bulls (n = 12 animals per treatment) for the respective journey (J) times pre- and post-transport.

Pre-transport			Post-transport			
**Journey** **(J)**	**-24 h**	**-0.25 h**	**0 h**	**4 h**	**12 h**	**24 h**

J 0	370.5 ± 49.8	373.2 ± 48.5	352.8 ± 45.8	360.1 ± 48.4	366.8 ± 48.9	356.9 ± 46.5
J 6	370.8 ± 32.6	367.1 ± 31.8	349.8 ± 29.6	362.2 ± 30.1	363.2 ± 32.6	362.2 ± 33.4
J 9	369.3 ± 50.1	362.4 ± 50.9	346.2 ± 48.2	359.2 ± 48.0	355.2 ± 48.9	361.2 ± 50.0
J 12	367.7 ± 26.2	367.0 ± 26.6	346.1 ± 26.1	360.8 ± 27.1	359.7 ± 27.1	365.8 ± 27.5
J 18	365.2 ± 27.0^a^	363.8 ± 26.3^a^	339.5 ± 22.7^b^	345.6 ± 24.0^ab^	350.1 ± 24.9^ab^	349.3 ± 25.8^ab^
J 24	362.8 ± 21.1^a^	359.7 ± 20.7^a^	332.6 ±19.6^b^	346.4 ± 19.5^ab^	354.7 ± 22.2^ab^	352.7 ± 21.2^ab^

Control = J 0 not transported; animals transported for journey (J) durations of 6, 9, 12, 18 and 24 hours (h) by road at a stocking density of 1.02 m^2 ^per animal. ^a,b,^within a row, means differ from pre-transport baseline (-0.25 h pre-transport) at P < 0.05.

### Rectal temperature data

There was no effect (P > 0.05) of transport on rectal temperature (data not shown).

### Physiological, metabolic and haematological variables

In control animals (J 0), there was no change (P > 0.05) in albumin concentration (Table 4). There were transient increases in albumin concentrations in animals transported for 6 h (J 6) at 0 h post-transport (P < 0.05) and in animals transported for 9 h (J 9) at 0 h and 1 h post-transport compared with pre-transport baselines. Animals transported for 12 h (J 12) had greater (P < 0.05) albumin concentrations at 0 h, 1 h and 2 h post-transport compared with baseline. Following transportation of animals for 18 h (J 18) albumin concentrations were greater (P < 0.05) at 0 h and 1 h post-transport, whereas the J 24 animals had greater concentrations (P < 0.05) at 0 h, 1 h and 2 h post-transport.

**Table 4:** Treatment means (n = 12 animals per treatment) with SE^1 ^for plasma albumin, globulin and total protein pre (-0.25 h) and post-transport (0, 1, 2, 4, 6, 8, 12 and 24 h).

Transport	-0.25 h pre	0 h post	1 h post	2 h post	4 h post	6 h post	8 h post	12 h post	24 h post	SE^1^	Effect of time^2^
Albumin (g/L)											
J 0	26.4	27.1	27.3	26.8	27.3	27.3	27.1	27.6	26.9	0.57	P =0.95
J 6	27.7^a^	29.6^b^	29.4^ab^	29.2^ab^	29.1^ab^	28.5^ab^	27.6^ab^	27.8^ab^	27.5^ab^	0.64	P =0.10
J 9	27.1^a^	29.3^b^	29.11^b^	28.4^ab^	27.7^ab^	27.6^ab^	27.7^ab^	27.5^ab^	27.6^ab^	0.68	P= 0.28
J 12	27.8^a^	29.8^b^	30.0^b^	29.5^b^	28.8^ab^	28.3^ab^	28.6^ab^	28.3^ab^	28.5^ab^	0.43	P = 0.01
J 18	27.3^a^	29.7^b^	29.3^b^	28.7^ab^	28.9^ab^	28.7^ab^	28.5^ab^	28.3^ab^	27.4^ab^	0.58	P = 0.08
J 24	27.8^b^	29.7^b^	29.9^b^	30.2^b^	28.9^ab^	28.8^ab^	28.9^ab^	28.4^ab^	28.3^ab^	0.61	P = 0.10
Globulin (g/L)											
J 0	40.0	41.4	40.2	40.6	40.6	41.0	40.0	41.6	41.3	0.86	P = 0.85
J 6	39.2	41.1	41.0	39.9	39.0	38.6	38.8	38.4	37.8	0.93	P = 0.18
J 9	40.3	43.2	42.6	41.9	40.3	39.7	40.1	40.7	41.0	1.43	P = 0.68
J 12	37.6^a^	41.3^b^	41.0^b^	40.1^ab^	39.0^ab^	38.7^ab^	38.9^ab^	38.9^ab^	39.4^ab^	0.98	P = 0.19
J 18	39.9	43.4	42.6	42.6	42.2	41.5	41.6	41.8	40.7	1.79	P = 0.95
J 24	39.3^a^	43.1^b^	43.1^b^	43.2^b^	41.7^b^	40.5^ab^	41.1^ab^	40.9^ab^	40.7^ab^	0.81	P = 0.01
Total protein (g/L)											
J 0	66.4	68.5	67.5	67.5	67.9	68.4	67.1	69.1	68.2	1.07	P = 0.79
J 6	66.9^a^	70.6^b^	70.4^b^	69.2^ab^	68.3^ab^	67.1^ab^	66.4^ab^	66.2^ab^	65.3^ab^	0.88	P = 0.0001
J 9	67.4^a^	72.5^b^	71.7^b^	70.3^ab^	67.9^ab^	67.3^ab^	67.8^ab^	68.2^ab^	68.6^ab^	1.18	P = 0.01
J 12	65.4^a^	71.1^b^	70.9^b^	69.5^b^	67.7^ab^	67.0^ab^	67.4^ab^	67.2^ab^	67.8^ab^	1.00	P = 0.001
J 18	67.14^a^	73.0^b^	71.9^b^	71.4^ab^	71.0^ab^	70.2^ab^	70.1^ab^	70.1^ab^	68.2^ab^	1.61	P = 0.27
J 24	67.1^a^	72.8^b^	73.0^b^	73.4^b^	70.6^b^	69.3^ab^	70.0^b^	69.3^ab^	69.0^ab^	0.93	P = 0.0001

Control = J 0 not transported; animals transported for journey (J) durations of 6, 9, 12, 18 and 24 hours (h) by road at a stocking density of 1.02 m^2 ^per animal. ^a,b,^within a row, means differ from pre-transport baseline (-0.25 h pre-transport) at P < 0.05. ^1^Standard error (SE). ^2^Effect of time (exact P value).

Globulin concentrations were unchanged (P > 0.05) (Table 4) in non-transported control animals (J 0), and in animals transported for 6 (J 6), 9 (J 9) and 18 h (J 18) compared with their pre-transport baselines. There were transient increases in globulin concentrations in the J 12 treatment at 0 h and 1 h post-transport, compared with baseline (P < 0.05), whereas the J 24 treatment had greater (P < 0.05) globulin concentrations at 0 h, 1 h, 2 h and 4 h post-transport compared with baseline.

In non-transported control animals (J 0), there was no change (P > 0.05) in total protein concentration (Table 4). There were transient increases in protein concentrations for the 6 h (J 6) and 9 h (J 9) treatments at 0 h and 1 h post-transport compared with their pre-transport baselines (P < 0.05). Animals transported for 12 h (J 12) had greater (P < 0.05) total protein concentrations at 0 h, 1 h and 2 h post-transport compared with baseline. Following transportation of animals for 18 h (J 18) total protein concentrations were greater (P < 0.05) at 0 h and 1 h post-transport compared with pre-transport values whereas protein concentrations were greater for the 24 h (J 24) treatment at 0 h, 1 h, 2 h, 4 h and 8 h (P < 0.05) post-transport compared with baseline.

In the non-transported controls (J 0), J 18 and J 24 treatments, urea concentrations were greater (P < 0.05) than baseline at 0 h to 12 h and were not different (P > 0.05) at 24 h (Table 5). Animals transported for 6 h (J 6) had greater (P < 0.05) urea concentrations at 0 h to 12 h post-transport while the J 9 treatment had greater (P < 0.05) urea concentrations at 0 h, 1 h and 2 h post-transport compared with pre-transport baselines. Animals transported for 12 h (J 12) had greater (P < 0.05) urea concentrations at 0 h to 8 h post-transport compared with baseline.

**Table 5:** Treatment means (n = 12 animals per treatment) with SE^1 ^for plasma urea, βHB and creatine kinase activity pre (-0.25 h) and post-transport (0, 1, 2, 4, 6, 8, 12 and 24 h).

Transport	-0.25 h pre	0 h post	1 h post	2 h post	4 h post	6 h post	8 h post	12 h post	24 h post	SE^1^	Effect of time^2^
Urea (mmol/L)											
J 0	2.3^a^	4.1^b^	4.1^b^	4.3^b^	4.6^b^	4.6^b^	4.3^b^	3.5^b^	2.9^ab^	0.29	P = 0.001
J 6	2.4^a^	2.9^b^	3.0^b^	3.2^b^	3.7^b^	4.2^b^	4.5^b^	4.4^b^	3.6^ab^	0.14	P = 0.001
J 9	3.2^a^	3.5^b^	3.6^b^	3.9^b^	4.1^ab^	4.4^ab^	4.3^ab^	3.7^ab^	2.9^ab^	0.25	P = 0.001
J 12	3.3^a^	4.1^b^	4.3^b^	4.5^b^	4.8^b^	4.7^b^	4.2^b^	3.5^ab^	2.6^ab^	0.19	P = 0.001
J 18	2.2^a^	4.3^b^	4.5^b^	4.6^b^	4.8^b^	4.5^b^	4.0^b^	3.2^b^	2.3^ab^	0.17	P = 0.001
J 24	2.4^a^	5.4^b^	5.4^b^	5.5^b^	5.7^b^	5.7^b^	5.4^b^	4.3^b^	2.8^ab^	0.27	P = 0.001
βHB (mmol/L)											
J 0	0.18^a^	0.22^ab^	0.20^ab^	0.19^ab^	0.25^ab^	0.29^b^	0.36^b^	0.28^b^	0.29^b^	0.03	P = 0.001
J 6	0.17^a^	0.16^ab^	0.17^ab^	0.22^b^	0.25^b^	0.25	0.25^b^	0.24^b^	0.26^b^	0.01	P = 0.001
J 9	0.18^a^	0.16^ab^	0.19^ab^	0.25^b^	0.31^b^	0.32^b^	0.30^b^	0.28^b^	0.30^b^	0.02	P = 0.001
J 12	0.20^a^	0.16^ab^	0.17^ab^	0.23^ab^	0.28^b^	0.30^b^	0.29^b^	0.25^b^	0.32^b^	0.15	P = 0.001
J 18	0.18^b^	0.20^ab^	0.14^ab^	0.18^ab^	0.20^ab^	0.28^b^	0.27^b^	0.28^b^	0.31^b^	0.02	P = 0.001
J 24	0.20^a^	0.18^ab^	0.14^ab^	0.17^ab^	0.22^ab^	0.27^ab^	0.36^b^	0.39^b^	0.34^b^	0.20	P = 0.001
CK (U/L)											
J 0	159.6^a^	437.1^b^	511.4^b^	551.4^b^	552.4^b^	524.8^b^	483.6^b^	430.4^ab^	381.0^ab^	60.3	P = 0.86
J 6	160.6^a^	377.9^ab^	443.9^ab^	521.1^ab^	627.0^b^	691.4^b^	605.6 ^b^	487.42^b^	427.2^ab^	69.5	P = 0.44
J 9	242.2^a^	428.3^ab^	522.7^b^	469.7^ab^	511.1^ab^	469.0^ab^	357.4^ab^	364.5^ab^	319.6^ab^	286.0	P = 0.53
J 12	187.7^a^	368.5^ab^	398.2^ab^	548.3^b^	545.0^b^	497.3^b^	494.8^b^	431.7^b^	404.6^ab^	77.5	P = 0.04
J 18	159.6^a^	472.9^ab^	497.1^ab^	467.1^b^	445.3^b^	477.9^b^	475.7^b^	437.5^b^	393.4^ab^	124.0	P = 0.17
J 24	162.3^a^	496.20^b^	545.7^b^	598.6^b^	598.7^b^	600.0^b^	631.6^b^	552.0^b^	340.3^ab^	92.8	P = 0.02

Control = J 0 not transported; animals transported for journey (J) durations of 6, 9, 12, 18 and 24 hours (h) by road at a stocking density of 1.02 m^2 ^per animal. ^a,b,^within a row, means differ from pre-transport baseline (-0.25 h pre-transport) at P < 0.05. ^1^Standard error (SE). ^2^Effect of time (exact P value).

βHB concentrations were greater (P < 0.05) at 6 h to 24 h following the end of the 24 h experimental period in non-transported control animals (J 0), when compared with baseline concentrations (Table 5). Animals transported for 6 h (J 6) and 9 h (J 9) had greater (P < 0.05) βHB concentrations than baseline at 2 h to 24 h post-transport, whereas, animals transported for 12 h (J 12) had greater (P < 0.05) βHB concentrations than baseline at 4 h to 24 h post-transport. Following transportation of animals for 18 h (J 18) βHB concentrations were greater (P < 0.05) than baseline at 6 h to 24 h and animals transported for 24 h had greater (P < 0.05) βHB concentrations than baseline at 8 h, 12 h and 24 h post-transport compared with baseline.

CK activities were greater (P < 0.05) than baseline in non-transported control animals (J 0) at 0 h to 8 h post the 24 h experimental rest period (Table 5). Post-transport, CK activities were greater (P < 0.05) than baseline for the J 6 treatment at 4 h to 12 h, for the J 9 treatment at 1 h post-transport and for the J 12 treatment at 2 h to 8 h post-transport. Animals transported for 18 h (J 18) had greater (P < 0.05) CK activities than baseline at 2 h to 12 h post-transport whereas levels were greater (P < 0.05) for the 24 h treatment at 0 h to 12 h post-transport compared with baseline.

Haptoglobin concentrations were greater (P < 0.05) than baseline in non-transported control animals at 24 h following the end of the experimental 24 h rest period (Table 6), and were unchanged in animals transported for 6 (J 6) and 9 h (J 9). There were transient increases (P < 0.05) in haptoglobin concentrations for the J 12 treatment at 1 h, 12 and 24 h post-transport, for the J 18 treatment at 24 h post-transport and for the J 24 treatment at 6 to 24 h post-transport compared with baseline.

**Table 6:** Treatment means (n = 12 animals per treatment) with SE^1 ^for plasma haptoglobin, glucose and NEFA pre (-0.25 h) and post-transport (0, 1, 2, 4, 6, 8, 12 and 24 h).

Transport	-0.25 h pre	0 h post	1 h post	2 h post	4 h post	6 h post	8 h post	12 h post	24 h post	SE^1^	Effect of time^2^
Haptoglobin (Hgb binding capacity/L)											
J 0	0.16 ^a^	0.34^ab^	0.34^ab^	0.32^ab^	0.34^ab^	0.32^ab^	0.33^ab^	0.36^ab^	0.60^b^	0.09	P = 0.19
J 6	0.10	0.08	0.09	0.10	0.12	0.11	0.09	0.11	0.16	0.01	P = 0.0002
J 9	0.15	0.16	0.16	0.15	0.15	0.18	0.19	0.22	0.29	0.08	P = 0.96
J 12	0.10^a^	0.14^ab^	0.17^b^	0.14^ab^	0.13^ab^	0.13^ab^	0.14^ab^	0.17^b^	0.23^b^	0.02	P = 0.013
J 18	0.09^a^	0.16^ab^	0.15^ab^	0.13^ab^	0.14^ab^	0.14^ab^	0.14^ab^	0.14^ab^	0.26^b^	0.03	P = 0.03
J 24	0.05^a^	0.12^ab^	0.13^ab^	0.12^ab^	0.11^ab^	0.14^b^	0.24^b^	0.25^b^	0.41^b^	0.03	P = 0.0001
Glucose (mmol/L)											
J 0	4.7^a^	4.1^b^	4.3^ab^	4.3^ab^	3.8^b^	4.0^b^	4.0^b^	4.1^b^	4.4^ab^	0.14	P = 0.002
J 6	4.7^a^	4.9^ab^	5.4^b^	5.4^b^	4.9^ab^	4.9^ab^	4.9^ab^	4.9^ab^	4.2^ab^	0.22	P = 0.01
J 9	4.4^a^	5.7^b^	5.5^b^	5.2^b^	4.7^ab^	4.6^ab^	4.4^ab^	4.3^ab^	4.3^ab^	0.15	P = 0.0001
J 12	4.8^a^	6.3^b^	6.0^b^	5.5^b^	5.3^ab^	4.9^ab^	4.8^ab^	4.6^ab^	4.7^ab^	0.20	P = 0.0001
J 18	4.7^a^	5.7^b^	5.9^b^	5.1^ab^	4.7^ab^	4.5^ab^	4.5^ab^	4.2^ab^	4.4^ab^	0.22	P = 0.0001
J 24	4.3^a^	5.8^b^	5.4^b^	5.1^b^	4.8^ab^	4.6^ab^	4.2^ab^	4.1^ab^	4.2^ab^	0.19	P = 0.0001
NEFA (μmol/L)											
J 0	0.25^a^	0.88^b^	0.69^b^	0.42^b^	0.34^ab^	0.32^ab^	0.29^ab^	0.22^ab^	0.56^b^	0.05	P = 0.0001
J 6	0.26^a^	0.35^ab^	0.19^ab^	0.14^ab^	0.15^ab^	0.18^ab^	0.25^ab^	0.42^b^	0.23^ab^	0.04	P = 0.0001
J 9	0.24^a^	0.32^ab^	0.12^b^	0.09^b^	0.10^b^	0.13^b^	0.15^ab^	0.22^ab^	0.22^ab^	0.03	P = 0.0001
J 12	0.28^a^	0.32^ab^	0.12^b^	0.11^b^	0.12^b^	0.15^b^	0.21^ab^	0.21^ab^	0.35^ab^	0.03	P = 0.0001
J 18	0.19^a^	0.40^b^	0.17^ab^	0.16^ab^	0.23^ab^	0.30^ab^	0.25^ab^	0.30^ab^	0.55^b^	0.06	P = 0.0001
J 24	0.27^a^	0.44^b^	0.40^b^	0.25^ab^	0.22^ab^	0.22^ab^	0.15^ab^	0.13^ab^	0.48^b^	0.04	P = 0.0001

Control = J 0 not transported; animals transported for journey (J) durations of 6, 9, 12, 18 and 24 hours (h) by road at a stocking density of 1.02 m^2 ^per animal. ^a,b,^within a row, means differ from pre-transport baseline (-0.25 h pre-transport) at P < 0.05. ^1^Standard error (SE). ^2^Effect of time (exact P value).

In non-transported control animals (J 0), glucose concentrations were lower (P < 0.05) than baseline at 0 h, 4 h, 6 h, 8 h and 12 h relative to the end of the initial 24 h experimental period (Table 6). There were transient increases in glucose concentrations for the J 6 treatment at 1 h and 2 h post-transport, for the J 9, J 12 and J 24 treatments 0 h, 1 h and 2 h post-transport compared with baseline, whereas the J 18 treatment had greater glucose concentrations (P < 0.05) at 0 h and 1 h post-transport.

NEFA concentrations were greater in non-transported control animals (J 0) at 0 h, 1 h, 2, h and 24 h compared with baseline (Table 6). There were transient increases (P < 0.05) in NEFA concentrations for the J 6 treatment at 12 h post-transport whereas the J 9 and J 12 treatments had lower (P < 0.05) NEFA concentrations at 1 h to 6 h post-transport. Following transportation of animals for 18 h (J 18), NEFA concentrations were greater (P < 0.05) at 0 h and 24 h whereas animals transported for 24 h had greater concentrations than baseline at 0 h, 1 h and 24 h post-transport.

WBC numbers were greater (P < 0.05) than baseline at 4 h to 24 h (Table 7) in non-transported control animals (J 0). There were transient increases in WBC number for the J 6 treatment at 0 h to 24 h post-transport and for the J 9 and J 18 treatments at 0 h to 12 h post-transport, while the J 12 and J 24 treatments had greater (P < 0.05) WBC number at 0 h to 24 h post-transport.

**Table 7:** Treatment means (n = 12 animals per treatment) with SE^1 ^for white blood cell (WBC) number and the neutrophil:lymphocyte (N:L) ratio pre (-0.25 h) and post-transport (0, 1, 2, 4, 6, 8, 12 and 24 h).

Transport	-0.25 h pre	0 h post	1 h post	2 h post	4 h post	6 h post	8 h post	12 h post	24 h post	SE^1^	Effect of time^2^
WBC (× 10^3^/μL)											
J 0	9.2^a^	9.1^ab^	8.9^ab^	9.8^ab^	11.5^b^	11.3^b^	11.9^b^	12.6^b^	12.2^b^	0.72	P = 0.0003
J 6	8.9^a^	14.7^b^	15.2^b^	15.1^b^	14.4^b^	14.5^b^	14.2^b^	13.5^b^	11.8^b^	0.07	P = 0.001
J 9	9.7^a^	15.3^b^	15.7^b^	15.5^b^	15.2^b^	15.1^b^	15.1^b^	13.8^b^	11.7^ab^	0.79	P = 0.0001
J 12	9.3^a^	12.7^ab^	13.2^b^	13.3^b^	13.2^b^	13.3^b^	13.7^b^	12.5^b^	11.6^b^	0.76	P = 0.003
J 18	10.4^a^	13.1^b^	13.1^b^	13.4^b^	13.1^b^	14.0^b^	14.1^b^	13.9^b^	12.2^ab^	0.65	P = 0.004
J 24	10.4^a^	11.9^b^	12.7 ^b^	12.2^b^	12.6^b^	13.3^b^	14.1^b^	13.4^b^	14.0^b^	0.99	P = 0.20
N:L ratio											
J 0	0.35^a^	0.28^ab^	0.32^ab^	0.40^ab^	0.51^ab^	0.68^ab^	0.77^ab^	1.35^b^	0.57^ab^	0.09	P = 0.0001
J 6	0.62^a^	1.30^b^	1.27^b^	1.33^b^	1.37^b^	1.20^ab^	1.15^ab^	0.78^ab^	0.76^ab^	0.05	P = 0.0001
J 9	0.44 ^a^	1.65 ^b^	1.63^b^	1.53^b^	1.13^b^	1.09^b^	1.12 ^b^	0.97^ab^	0.85^ab^	0.10	P = 0.0001
J 12	0.31^a^	1.15 ^b^	1.17^b^	1.12^b^	0.72^ab^	0.73^ab^	0.99^b^	0.93^b^	0.58^ab^	0.08	P = 0.0001
J 18	0.44^a^	0.95^ab^	1.07^b^	1.06^b^	1.01^b^	0.92^ab^	1.36^b^	1.05^b^	0.68^ab^	0.11	P = 0.0001
J 24	0.43^a^	0.99^b^	0.81^ab^	1.08^b^	1.10^b^	0.77^ab^	0.83^ab^	0.92^b^	0.84^ab^	0.14	P = 0.0001

Control = J 0 not transported; animals transported for journey (J) durations of 6, 9, 12, 18 and 24 hours (h) by road at a stocking density of 1.02 m^2 ^per animal. ^a,b,^within a row, means differ from pre-transport baseline (-0.25 h pre-transport) at P < 0.05. ^1^Standard error (SE). ^2^Effect of time (exact P value).

The N:L ratio was greater (P < 0.05) than baseline at 12 h (Table 7) in non-transported control animals (J 0). There were transient increases (P < 0.05) in N:L ratios for the J 6 treatment at 0 h to 4 h post-transport; the J 9 treatment at 0 h to 8 h post-transport; the J 12 treatment at 0 h to 2 h and 8 and 12 h post-transport; the J 18 treatment at 1 h to 4 h, and 8 h and 12 h post-transport and the J 24 treatment at 0 h, 2 h, 4 h and 12 h post-transport.

In non-transported control animals, lymphocyte percentage was lower (P < 0.05) and neutrophil percentage was greater (P < 0.05) than baseline at 6 h to 24 h following the end of the 24 h experimental period (Table 8). Animals transported for 6 (J 6), 9 (J 9), 12 (J 12), 18 (J 18) and 24 h (J 24) had lower (P < 0.05) lymphocyte percentage and greater (P < 0.05) neutrophil percentage than baseline at 0 h to 24 h post-transport.

**Table 8:** Treatment means (n = 12 animals per treatment) with SE^1 ^for lymphocyte and neutrophil percentage (%) pre (-0.25 h) and post-transport (0, 1, 2, 4, 6, 8, 12 and 24 h).

Transport	-0.25 h pre	0 h post	1 h post	2 h post	4 h post	6 h post	8 h post	12 h post	24 h post	SE^1^	Effect of time^2^
Lymphocyte (%)											
J 0	73.2^a^	77.8^ab^	74.5^ab^	71.9^ab^	68.3^ab^	62.0^b^	58.3^b^	44.8^b^	63.8^b^	3.35	P = 0.0001
J 6	65.2^a^	43.6^b^	46.7^b^	43.1^b^	42.9^b^	46.3^b^	47.2^b^	56.0^b^	57.3^b^	2.49	P = 0.0001
J 9	65.9^a^	39.8^b^	41.4^b^	41.8^b^	47.7 ^b^	48.4^b^	47.8^b^	52.0^b^	53.7^b^	3.02	P = 0.0001
J 12	74.6^a^	49.5^b^	46.5^b^	49.0^b^	59.5^b^	60.0^b^	51.0^b^	51.6^b^	63.6^b^	2.99	P = 0.0001
J 18	68.4^a^	52.8^b^	48.9^b^	50.3^b^	50.8^b^	51.8^b^	43.5^b^	49.6^b^	59.8^b^	2.94	P = 0.0001
J 24	69.4^a^	50.8^b^	56.6^b^	49.2^b^	47.2^b^	56.1^b^	53.8^b^	52.2^b^	56.3^b^	2.79	P = 0.0001
Neutrophil (%)											
J 0	24.1^a^	19.8^b^	23.1^b^	26.6^b^	29.9^b^	36.1^b^	40.3^b^	54.1^b^	34.8^b^	3.31	P = 0.0001
J 6	30.8^a^	55.2^b^	55.5^ab^	55.6^ab^	56.1^ab^	52.8^b^	51.3^b^	42.3^b^	42.8^b^	2.56	P = 0.0001
J 9	25.6^a^	60.1^b^	59.1^b^	57.9^b^	51.8^b^	51.0^b^	50.5^b^	45.8^b^	42.8^b^	2.74	P = 0.0001
J 12	22.9^a^	48.6^b^	52.8^b^	50.8^b^	39.9^b^	38.7^b^	47.5^b^	46.4^b^	34.7^b^	3.04	P = 0.0001
J 18	28.2^a^	46.1^b^	50.4^b^	48.7^b^	48.7^b^	44.8^b^	54.5^b^	46.7^b^	36.8^b^	2.96	P = 0.0001
J 24	28.9^a^	48.7^b^	42.7^b^	49.0^b^	50.2^b^	41.3^b^	42.3^b^	43.6^b^	41.8^b^	2.90	P = 0.0001

Control = J 0 not transported; animals transported for journey (J) durations of 6, 9, 12, 18 and 24 hours (h) by road at a stocking density of 1.02 m^2 ^per animal. ^a,b,^within a row, means differ from pre-transport baseline (-0.25 h pre-transport) at P < 0.05. ^1^Standard error (SE). ^2^Effect of time (exact P value).

RBC numbers were lower (P < 0.05) than baseline in non-transported control animals at 0 h to 24 h relative to the end of the first 24 h experimental period (Table 9). There were no differences (P > 0.05) in RBC numbers in the J 6, J 9, J 12, J 18 and J 24 treatments following transport compared with baseline values.

**Table 9:** Treatment means with SE^1 ^for red blood cell (RBC) number, whole blood haemoglobin (Hgb) concentrations, and haematocrit (%) pre (-0.25 h) and post-transport (0, 1, 2, 4, 6, 8, 12 and 24 h).

Transport	-0.25 h pre	0 h post	1 h post	2 h post	4 h post	6 h post	8 h post	12 h post	24 h post	SE^1^	Effect of time^2^
RBC (× 10^6^/μL)											
J 0	10.0^a^	9.3^b^	8.8^b^	8.8^b^	9.1^b^	9.3^b^	8.8^b^	9.1^b^	9.0^b^	0.25	P = 0.02
J 6	9.4	9.5	9.5	9.4	9.2	9.2	9.0	9.1	8.7	0.30	P = 0.58
J 9	9.3	9.6	9.6	9.5	9.3	9.2	9.3	9.4	9.1	0.40	P = 0.993
J 12	9.6	9.5	9.7	9.8	9.6	9.4	9.4	9.4	9.0	0.30	P = 0.823
J 18	9.9	9.8	9.8	9.8	10.1	9.8	9.9	10.0	9.2	0.29	P = 0.75
J 24	8.8	8.9	9.1	9.2	9.0	8.7	8.8	8.9	8.7	0.25	P = 0.81
Hgb (g/dL)											
J 0	11.2^a^	10.4^b^	10.0^b^	9.8^b^	10.1^b^	10.2^b^	9.7^b^	9.9^b^	9.9^b^	0.28	P = 0.01
J 6	10.0	10.0	10.0	10.0	9.7	9.6	9.5	9.6	9.3	0.24	P = 0.35
J 9	9.5	9.5	9.7	9.5	9.3	9.3	9.2	9.6	9.2	0.26	P = 0.887
J 12	9.9^a^	9.8^ab^	10.1^ab^	10.1^ab^	9.9^ab^	9.7^ab^	9.9^ab^	9.8^ab^	9.3^b^	0.18	P = 0.120
J 18	10.1	10.0	10.1	10.0	10.1	9.9	9.8	10.1	9.1	0.26	P = 0.15
J 24	9.0	9.1	9.4	9.5	9.2	9.2	9.3	9.3	9.3	0.22	P = 0.86
Haematocrit (%)											
J 0	33.9^a^	31.7^b^	29.8^b^	29.5^b^	30.3^b^	30.3^b^	29.1^b^	30.2^b^	29.7^b^	0.94	P = 0.02
J 6	30.4^a^	31.2^ab^	31.1^ab^	30.8^ab^	29.9^ab^	29.7^ab^	29.2^ab^	29.5^ab^	27.6^b^	0.78	P = 0.05
J 9	28.5	29.7	29.8	29.2	28.7	28.3	28.5	28.9	27.7	0.94	P = 0.87
J 12	29.9^a^	30.0^ab^	30.4^ab^	30.4^ab^	29.7^ab^	29.1^ab^	29.1^ab^	29.0^ab^	27.9^b^	0.66	P = 0.20
J 18	30.1	29.9	30.1	29.5	30.6	29.8	29.8	30.5	28.0	0.88	P = 0.73
J 24	27.2	27.4	27.8	28.3	27.3	27.1	27.1	27.6	27.0	0.65	P = 0.93

Control = J 0 not transported; animals transported for journey (J) durations of 6, 9, 12, 18 and 24 hours (h) by road at a stocking density of 1.02 m^2 ^per animal. ^a,b,^within a row, means differ from pre-transport baseline (-0.25 h pre-transport) at P < 0.05. ^1^Standard error (SE). ^2^Effect of time (exact P value).

Haemoglobin (Hgb) concentrations were lower (P < 0.05) at 0 h to 24 h in non-transported control animals compared with baseline (Table 9). There were no differences (P > 0.05) in Hgb concentrations for J 6, J 9, J 18 and J 24 treatment groups following transport while animals transported for 12 h had lower Hgb concentrations (P < 0.05) than baseline at 24 h post-transport.

Haematocrit % was lower (P < 0.05) at 0 h to 24 h post-transport (Table 9) compared with baseline in non-transported control animals. Animals transported for 6 h (J 6) and 12 h (J 12) had lower (P < 0.05) haematocrit % than baseline at 24 h post-transport. Post-transport there were no differences (P > 0.05) in haematocrit % in J 9, J 18 and J 24 treatments compared with baseline values.

There was no difference (P > 0.05) in mean corpuscular volume (MCV) and MCHC following transport (data not shown). Platelet numbers were lower (P < 0.05) at 0 h (mean (s.d.) 424.3 (140.4) × 10^3^cells/μL) in non-transported control animals compared with 4 h (mean (s.d.) 536.7 (135.4) × 10^3^cells/μL), 6 h (mean (s.d.) 561.3 (155.8) × 10^3^cells/μL) and 12 h (mean 539.9 (s.d.) (124.0) × 10^3^cells/μL) and values returned to baseline at 24 h. Platelet numbers were not different (P > 0.05) post-transport for the J 6 to J 24 treatments (data not shown).

## Discussion

In the present study, the series of transport journeys (6 h to 24 h) by roads showed that transportation of Charolais beef bulls affected live weight, haematological and physiological measurements of metabolism and inflammation. The biological measures which were most sensitive to the stress of transport, on journeys of 6 h to 24 h duration, were total protein, urea, ßHB, glucose, NEFA, the acute phase protein (haptoglobin) and haematological variables (including WBC number, neutrophil and lymphocyte %). This was not unexpected as we reported similar responses in young bulls that were subjected to 8 h transport [4]. However, it also flags a cautionary note when drawing conclusions based on a single cohort of animals that to increase the probability of statistically significant results when measuring other variables (for example RBC number, albumin, globulin, and haemoglobin and creatine kinase activity) it may be necessary to increase the group size per treatment.

The present experiment was designed to minimize any possible bias that would affect the study, however, we accept that it is not possible to exclude all bias since all studies are affected by some degree of bias. In the present study, the bulls were habituated to housing for 100 days pre-transport and were fed the same diet. All of the bulls were naïve to transport and each journey was carried out singly over a 6-week period. For each journey, 12 bulls were assigned to two pens on the transporter with 6 animals per pen and each journey was made by the same driver using similar road conditions. The bulls were blood sampled by the same person and the same chute was used at each experimental blood collection time point. In the statistical analysis of the data, animal was the experimental or measurement unit.

The changes in live weight post-transport were transitory and may be attributed to a loss of gut-fill over the journeys and possibly due to mild dehydration, urination and fasting during the longer journeys [15-17,20,21]. The loss in live weight in control animals in the present study may be attributed to the change in diet, as silage and concentrates were withdrawn and animals had access to hay and water only for 24 h corresponding to the longest duration of transport. Rectal temperature was not changed during each of the respective transport journeys indicating that there was no clinical infection induced by transport and no evidence of clinical disease. Although rectal temperature was not changed, it is a well known indicator of an inflammatory response to infection in newly arrived feedlot calves [20]. The lack of an effect of transport on rectal body temperature may be related to the ambient temperature since animals would not have been exposed to extreme range of temperatures during the present series of journeys.

The development of electrolyte and acid-base imbalances has been reported in extended transport journeys where fasting has exceeded 2 days or more [22]. In the present study, transportation had transient effects on metabolism as demonstrated by significant changes in the plasma concentrations of total protein, urea, βHB, NEFA and glucose. Total protein concentrations increased with journey duration in transported animals, however, by 12 h post-transport concentrations had returned to pre-transport baseline levels. Transport stress has been reported to cause dehydration and may manifest itself as a hyperproteinemia [23]. The changes in protein concentrations reported in the present study post-transport are more likely the result of metabolic compensation for a mild metabolic acidosis secondary to water loss and feed deprivation during transport. These findings suggest that pre-transport mixing and transportation alters protein metabolism. Metabolic variables of protein, energy, and mineral metabolism in cattle as well as rumen function have been examined following transportation. Changes in circulating total protein, albumin, and urea, have been reported to increase following transportation [3,24]. Changes in energy metabolism as evidenced by an increases in blood glucose [1,16,17], lactate dehydrogenase, glutamic pyruvic transaminase, and glutamic oxalacetic transaminase [24], decreases in βHB [3], increased haematocrit % and plasma corticosteroid concentration [25] have been reported. When the body prepares to react to a potentially stressful situation an increase in energy metabolism may be precipitated [26].

Increases in plasma glucose concentrations are mainly due to glycogenolysis associated with the increase in circulating catecholamines and glucocorticoids which are released during the stress of transport [27]. Glucose levels returned to baseline in all treatments compared with baseline within 4 h of transport. Urea, NEFA and βHB concentrations were elevated in control and all transported animals and concentration remained greater than baseline for animals transported on journey durations ranging from 0 h to 24 h. Urea concentrations had returned to pre-transport baseline values by 24 h post-transport in all animals.

Physiological processes, such as the sleep-wake cycles, locomotor activity, body temperature, hormone secretion, and metabolism, are under the control of circadian clocks and are influenced by stress. Circadian control of glucose metabolism was recognized from early studies demonstrating variation in glucose tolerance and insulin action across the day [28,29]. Increased energy metabolism is a hallmark of the stress response as the body prepares to react to a potentially stressful situation. We have previously reported increases in several of these protein metabolites in response to transportation [3,4,17]. These differences may be due to a number of factors including the duration of the journey and that animals did not have access to feed during transportation. Additionally, increased circulating CK is an indication of muscular activity and or/bruising and is often measured in transported cattle as a measure of bruising [5]. Creatine and phosphocreatine are major intracellular solutes in muscle cells. Increases in plasma CK activity after different transport journeys have been described by different authors [16,19,30]. A direct relationship between the duration of transport and the rise in the activity of the enzyme has been reported [19]. Fasting has also been reported to increase the activity of the enzyme, and the rise could be masked by the high values obtained after transport [31]. In the present study, CK activities returned to pre-treatment baseline values within 12 h for all transported animals. Interestingly, control animals in the present study had elevated CK activity while the magnitude of the changes were small the return to baseline was rapid. Circulating creatine kinase activity is often measured in transported cattle as a measure of bruising [32], indicating that the bulls in the current study may have experienced some physical stress.

Changes in acute phase protein concentrations during transportation have been reported but the results are variable. Haptoglobin, an acute phase protein, is released by hepatocytes and mediate the inflammatory response to injury, trauma, or infection [33]. The presence of acute phase proteins in the circulation may be an excellent biomarker of inflammation as they are readily measurable in serum or plasma and may even discriminate between acute and chronic inflammation in cattle [34]. Acute phase proteins are present in very low concentrations in plasma and increase in concentration following tissue injury and inflammation [35,36]. In the present study haptoglobin concentrations were increased relative to the baseline in control and transported animals up to 24 h post-transport, with the exception of the animals transported for 6 h. Serum haptoglobin was reported to be elevated in calves transported for 2 days and levels were negatively correlated with lymphocyte function [37]. In a separate study transporting bulls at different stocking densities, plasma haptoglobin concentrations were unchanged, while plasma fibrinogen levels were reduced [3,4]. Fibrinogen, ceruloplasmin, serum amyloid-A, and α-acid glycoprotein were assayed in the plasma of transported and commingled calves and found to be increased post-transportation; however, haptoglobin concentrations were greater in non-transported versus transported calves [20].

Alterations in immunity are of great importance following transportation stress as these alterations are thought to be associated with increased incidence and severity of respiratory diseases. Many measures of immunological changes relate to immune cell numbers in the blood. Similar to the findings of the present study, most studies observe a leukocytosis that is marked by neutrophilia, which may occur in conjunction with a decrease in the number of other cells (lymphopaenia, eosinopaenia) [9,10,38]. Changes in the haematological responses of cattle to transport have been reported with increases in RBC number, haematocrit percentage and haemoglobin concentration following transportation of steers [17,39]. In the present study, all transported animals had greater neutrophil percentage and lower lymphocyte percentage post-transport. Haemoglobin concentrations and RBC numbers were within normal blood referenced ranges [40-43]. The neutrophilia observed in control animals is most likely due to the effect of stress related to the mixing and the handling procedures. Blood lymphocytes contain concentrations of glucocorticoid and adrenergic receptors [44], which are down-regulated in response to stress [45] and suggests that alterations in the blood cell composition of leukocytes may have an important role in the responsiveness of the immune system when stress challenged. There was no major change in haematocrit % compared with baseline in animals transported for 6 h to 24 h. Animals in the present study had *ad libitum *access to water on the transporter and they received the last feeding immediately before loading and these factors may have prevented the animals from showing signs of dehydration. Elevated haematocrit % has been reported following transport in association with greater erythrocyte counts in the circulation [17,25,46,47] and a significant increase in haematocrit values indicates mainly dehydration.

Measures of immunological changes relate to immune cell numbers in the blood and immune cell function. A number of studies have reported leukocytosis that is marked by neutrophilia, and which may be present with a decrease in the number of other cells (lymphopaenia, eosinopaenia) [8,10,17]. Bovine alveolar macrophages, isolated from bronchoalveolar lavage (BAL) fluid, have a reduced respiratory burst function after 4 h of transportation [48]. The respiratory burst function is necessary to produce reactive oxygen species that are toxic to phagocytosed pathogens, and these results may represent impaired lung defence. In contrast, enhanced respiratory burst activity has been found in neutrophils of transported calves [10]. Apoptosis of neutrophils in combination with increased migratory capacity in dairy cows have been reported after 4 h of transportation [49]. The normal referenced ranges for differential counts, neutrophils are in the range 15-45 [40,50]. Within the range of transport times analysed, there were no significant changes in MCV and MCHC values. In the present study, the changes in the composition of the blood cell variables reflect the physiological response of the bulls to the stress of mixing, fasting and/or transportation.

In the current study, several measures of physiological, metabolic and haematological variables were investigated in the plasma of bulls subjected to transport journeys ranging from 6 to 24 h durations. It is evident that transportation of bulls has effects on biomarkers of metabolism as demonstrated by significant changes in the plasma concentrations of protein, glucose and NEFA. Additionally, circulating creatine kinase activity is a useful measure which is often monitored in transported cattle as a measure of bruising. Changes in CK activity indicate that the bulls in the current study may have experienced some muscle damage and physical stress, particularly after the longer duration journeys. The acute phase protein, haptoglobin, is a useful biomarker of inflammation and together with changes in haematological cellular variables would suggest a pro-inflammatory state during transportation stress. The pronounced neutrophilia and lymphopenia following transportation observed in this study are in agreement with previously reported findings following a variety of stressors, including transport stress [8,10,17]. Taken together, these results indicate that transportation stress alters physiological measures of metabolism and haematology. Thus, a profile or pattern of multiple physiological, metabolic and haematological variables may provide the most effective marker of altered homeostasis to allow an assessment of an animal's response to transport.

## Conclusions

Conditional on the statistical power of the present study and assumptions about meaningful indicators and effect of size, there were both similarities and differences between control and transported animals, however the differences did not appear sufficiently large or prolonged over the duration of the study. This is a single transport study for each journey duration and most of the physiological, metabolic and haematological variables which changed as a consequence of transport had recovered to baseline values within 24 h of journey completion. Non-transported control animals showed similar biological effects on the physiological and haematological variables to transported animals.

An increased understanding of the mechanism of stress and physiological adaptation induced in animals by mixing, handling and transport will lead to a greater understanding of transportation stress. Additionally, the effective recovery of the animals after 6-24 hours transport can lead to an important implication to recommend a proper lairage time when animals are transported by road. The results of this study indicate that the provision of a rest period of up to 24 hours post-transport with animals having access to feed and water should be optimal for animals to recover to their physiological pre-transport baseline.

## Methods

### Care of animals

This study was conducted at Teagasc, Grange Beef Research Centre, Dunsany, County Meath, Ireland. All animal procedures performed in this study were conducted under experimental licence from the Irish Department of Health and Children in accordance with the Cruelty to Animals Act 1876 and the European Communities (Amendment of Cruelty to Animals Act 1876) Regulation 2002 and 2005.

### Animal diets and composition

Seventy two Charolais bulls (mean weight (s.d.) 367 (35) kg) had *ad libitum *access to grass silage (*in vitro *DM digestibility = 762 g/kg), supplemented with 2.0 kg (as fed) barley/soybean concentrate (crude protein = 114.5 kg DM) per animal per day at Teagasc, Grange, Beef Research Centre, Dunsany, Co. Meath. The animals were managed and housed 6/pen in a slatted floor shed at a space allowance of 2.5 m^2^/head/animal for a 100-day winter period prior to transport and are representative of the type of animals that are commonly transported under commercial conditions. Animals had free access to water which was available through nipple drinkers in their home pens.

### Transport vehicle and environmental conditions

The study was conducted in Spring over a 6 week period from the 18^th ^of February to the 29^th ^of March. All journeys were made at the same starting time (8:00 h) and with the same transporter, transport conditions and driver. A total of 5 separate transport journeys were made of duration 6, 9, 12, 18 and 24 h. All animals were naïve to transport. On the morning of each journey, the animals were loaded at 8:00 h into two fan-ventilated pens (n = 6 animals/pen) on the lower deck of an air suspension articulated transporter at a stocking density of 1.02 m^2 ^per animal, and transported by road. The floor of the pens on the transporter was deep bedded with cereal straw for each journey. The animals had access to hay and water was available through nipple drinkers on the transporter, similar to the type that the bulls were accustomed to in their home pens. Bulls travelled for 6 h (280 km), 9 h (435 km), 12 h (582 km), 18 h (902 km) and 24 h (1192 km) and during each journey, transport was by primary (30% of the journey) and secondary (70% of the journey) roads and different road types and surfaces were encountered, respectively. The transporter was fitted with sensors, located 1.2 m above the floor of each pen on the transporter, for measuring ambient temperature (°C), relative humidity (RH; %), carbon dioxide (CO_2; _ppm), hydrogen sulphide (H_2_S; ppm), ammonia (NH_3_; ppm) air velocity (m/s) and vapour density (g/m^3^) continuously during transport. The ambient temperature and relative humidity during transport were recorded continuously using TinyTalk dataloggers (Radionics, Dublin, Ireland). Environmental measurements, including gases (NH_3_, H_2_S, CO_2_), relative humidity (RH) and temperature (°C), were recorded using QRae (Shawcity Ltd., UK), Testo 175 and Testo 445 portable multifunction probes (Testo UK, Ltd), respectively.

### Treatment groups

Seventy two Charolais bulls (mean weight (s.d.) 367 (35) kg) were randomly assigned by weight to one of six journey (J) treatment times of 0 (no transport), 6 (J 6), 9 (J 9), 12 (J 12), 18 (J 18) and 24 (J 24) h transport (n = 12 animals/treatment) at a stocking density of 1.02 m^2^/bull. On the morning of the journey the bulls underwent social mixing, were moved from their home pens to a race with a chute to facilitate live weight recordings and blood sampling. The animals were blood sampled pre-transport by jugular venipuncture to provide baseline physiological levels and again after the journey. Blood samples were collected by jugular venipuncture before (-0.25 h), immediately after (0 h) and at 1 h, 2 h, 4 h, 6 h, 8 h, 12 h and 24 h relative to time 0 h. Bulls were weighed using an animal weighing scale (Titan, Cattlemaster, Ireland) that was located at the exit area of the chute and rectal temperature was taken before, -24 h, -0.25 h, immediately after, and at 4, 12 and 24 h relative to completion of the transport journey. The bulls were loaded onto the transporter and assigned to two pens on the transporter and remained in the same social groupings for the remainder of the study. The study animals were transported alone on the transporter. On completion of each of the transport journeys (J 6 - J 24) the bulls were returned for a 24 h rest period to novel pens (n = 2) with 6 animals/pen in the housing environment and had *ad libitum *access to water and grass silage supplemented with 2.0 kg barley/soybean concentrate.

Non-transported control animals (J 0) (n = 12) were moved to two novel straw bedded pens (6 animals/pen) in the housing environment and animals had access to hay and water during the duration of the 'experimental' period coinciding with the maximum transport duration (24 h). Afterwards, control animals (J 0) had *ad libitum *access to grass silage supplemented with 2.0 kg barley/soybean concentrate, water, and no hay for the 24 h period coinciding with the 24 h 'post-transport' period. Control animals (J 0) were blood sampled before assignment (at -0.25 h) to the novel pens, after 24 h, and at 1 h, 2 h, 4 h, 6 h, 8 h, 12 h and 24 h relative to the completion of the 24 h experimental period. Control animals were weighed -24 h, -0.25 h, + 24 h and 4, 12, and 24 h after completion of the 24 h experimental period.

### Water intake

Water consumption (litres) was recorded during transport and in the 24 h period post-transport. Flow metres were attached to the animal drinking containers and consumption was recorded on a pen basis in the housing environment and on the transporter.

### Rectal temperature

The rectal body temperature was monitored before and after transport using a digital thermometer (Jorgen Kruuse A/S; Model VT-801BWC Lot No 0701).

### Assay procedures for physiological and haematological variables

Heparinised blood samples were collected by jugular venipuncture and the plasma was separated by centrifugation at 1,600 × g at 8°C for 15, and subsequently stored at -20°C until assayed. Albumin, urea, globulin, total protein, β hydroxybutyrate (βHB), and creatine kinase (CK) were measured on an automated analyser (Olympus AU 400, Japan) using the reagents supplied by Olympus. Unclotted (EDTA-treated) whole-blood samples were collected by jugular venipuncture at the same time as the heparinised blood samples for haematological analysis. Red blood cell (RBC) number, white blood cell (WBC) number, differential WBC (percentage lymphocyte and neutrophil), haematocrit (HCT) percentage (%), haemoglobin (Hgb) concentration, mean corpuscular volume (MCV), mean corpuscular haemoglobin concentration (MCHC) and platelet numbers were determined for unclotted (K_3_-EDTA-treated) whole-blood samples with an automated cell counter (Celltac MEK-6108K; Nihon-Kohdon, Tokyo, Japan) within 1 h of blood sampling. Thin blood smears were also prepared on glass slides and stained using the haematology 3-step stain for differential WBC counts (Accralab, Biochemical Sciences; Fisher Scientific Company, Middletown, VA). Plasma haptoglobin concentration was determined as the haemoglobin binding capacity using an appropriate assay (TP801: Tridelta Development Ltd., Greystones, Ireland), which was previously validated [32] and was quantified using a spACE automated analyser (Alfa Wassermann, Inc., West Caldwell, NJ, USA) [33]. Whole blood samples collected in sodium citrate tubes (Vacuette, Cruinn Diagnostics Limited, Ireland) were separated by centrifugation (3000 × g at 8°C for 10 minutes) and the plasma frozen at -20° until assayed for fibrinogen using a commercial kit adapted for bovine plasma [51] (Randox Laboratories Ltd., Crumlin, Antrim, N. Ireland; catalogue No. GL2623) on an automated analyser (Hitachi 705, Boehringer Mannheim, Lewes, East Sussex, UK).

## Statistical analyses

Physiological and haematological data, live weight data, water intake data and rectal temperature measurements were subjected to repeated measures using the PROC MIXED procedure of SAS (Version 9.1, SAS Institute, Cary, NC). Animal was the experimental unit and was specified as a repeated measures effect, and the dependence within animal was modelled using an unstructured covariance structure. The power of the statistical test was taken into consideration and was defined as the probability that the test will reject a false null hypothesis (i.e. that it will not make a Type II error). As power increases, the chances of a Type II error decrease. The probability of a Type II error is referred to as the false negative rate (β), therefore power is equal to 1 - β [52]. We calculated that with a group size of 12 bulls/treatment that the study would have 80% and greater power for the variables that we measured, meaning that if there was a difference of this given magnitude there would be an 80% chance of correctly detecting it as statistically significant. As "n" the number of animals per treatment is the main determinant of power of test [52], the authors considered it crucial that adequate group sizes of animals were used, however, optimal sample size is ultimately a function of the underlying parameters in the study. In the present study, two pen replicates with 6 bulls/pen (total n = 12) were utilized for each transport journey. Using the measurements which were investigated in the present study, this sample size was deemed adequate without utilizing more animals than ethically necessary. Differences between means were tested using the PDIFF option in PROC MIXED in SAS. The PDIFF option calculates a separate probability value for each pair of means being compared. Means were considered significantly different at the P < 0.05 probability level [53].

## Competing interests

The authors declare that they have no competing interests.

## Authors' contributions

BE designed the study. BE, MM and DJP performed the experiments. BE analyzed the data and prepared the manuscript. All authors read and approved the final manuscript.
